# Polycyclic Aromatic Hydrocarbons in Polish Traditionally and Industrially Smoked Meats as an Element of Monitoring and PAH Reduction Strategies

**DOI:** 10.3390/foods14030350

**Published:** 2025-01-22

**Authors:** Jolanta Kowalska, Monika Stanisławek, Agnieszka Latoch, Agata Marzec, Sabina Galus, Hanna Kowalska, Marta Ciecierska

**Affiliations:** 1 Department of Food Engineering and Process Management, Institute of Food Sciences, Warsaw University of Life Sciences, Nowoursynowska 159 C St., 02-787 Warsaw, Poland; monikastan1983@tlen.pl (M.S.); agata_marzec@sggw.edu.pl (A.M.); sabina_galus@sggw.edu.pl (S.G.); hanna_kowalska@sggw.edu.pl (H.K.); 2 Department of Animal Food Technology, University of Life Sciences in Lublin, 8 Skromna St., 20-704 Lublin, Poland; agnieszka.latoch@up.lublin.pl; 3 Department of Food Technology and Evaluation, Institute of Food Sciences, Warsaw University of Life Sciences, Nowoursynowska 159 St., 02-787 Warsaw, Poland

**Keywords:** polycyclic aromatic hydrocarbons (PAHs), benzo(a)pyrene, smoked meat products, traditional smoking, industrial smoking, monitoring, reduction strategies, GC-MS

## Abstract

This work aimed to analyze the PAH content in products smoked in traditional smokehouses with direct and indirect heat sources and in an industrial way as an element of PAH content monitoring in Polish market products. This research material comprised 12 smoked meats (W) and 38 sausages (K), medium or coarsely minced. The content of benzo(a)pyrene and the total content of four marker PAHs was determined by GC-MS. The analysis showed a significantly higher level of PAH contamination in products smoked using traditional methods. The results also indicate that the natural casing is not a barrier against PAH contamination during traditional smoking, and a higher degree of meat fragmentation, together with a small cross-section, increases the PAH content in this technological group. Concentrations of benzo(a)pyrene exceeding the permissible levels were found in the sausages smoked for more than 60 min. As part of the strategies for reducing the PAH content, among others, changing the furnace to an indirect one, shortening the time, lowering the smoking temperature, using artificial casings or removing casings before consumption, drying the product surface before the smoking process, using seasoned and bark-free wood, as well as additional smokehouse equipment, are recommended.

## 1. Introduction

Meat and meat products are important components of a healthy and balanced diet due to their high biological value, protein content, minerals, vitamins, and microelements [1,2].

Smoking is one of the oldest methods commonly used for food preservation, and it allows food to obtain characteristic organoleptic features, mainly taste, smell, and color. In addition, smokehouse smoke shows antibacterial and antioxidant qualities. Aside from the desirable qualities, the smoking process also generates substances that are undesirable regarding health and safety, like polycyclic aromatic hydrocarbons (PAHs). Smoking, baking, grilling, or barbecuing are mentioned primarily among different types of heat treatment that significantly contribute to PAH formation, resulting from incomplete wood combustion (pyrolysis) [1,2,3,4,5,6].

PAHs are compounds commonly found in the environment, but it is estimated that the main factor of human exposure to PAHs is the food we consume [5,7]. Polyarenes display cytotoxic, genotoxic, immunotoxic, teratogenic, carcinogenic, and mutagenic activity [1,5,8]. Research confirming the carcinogenic activity of PAHs was one of the arguments for implementing Commission Regulation (EU) No. 1881/2006 [9], which was then amended by Commission Regulation (EU) No. 915/2023, that set maximum levels of PAHs in meat and meat products. These regulations allow 2 μg/kg of benzo(a)pyrene and 12 μg/kg of the sum of four marker PAHs [10]. Nowadays, producers and consumers are interested in traditional food and food processed traditionally. In the case of traditional foods and traditionally smoked foods, the safety and quality of the product depend mainly on the skill of the smoker and his ability to control the smoking conditions [11,12].

Obtaining acceptable levels of PAHs in traditional or traditionally smoked products is very difficult and requires changes in technological practices. This, in turn, causes a deterioration in the sensory quality of smoked products. Therefore, derogations from the permitted levels of PAHs have been introduced for traditionally smoked products, provided these products are reported to the district veterinarian [13]. The derogation specified in the regulation of the Polish Minister of Agriculture and Rural Development on veterinary requirements in the production of cold cuts in the scope of permissible levels of contamination with PAHs specifies the maximum limits of benzo(a)pyrene and the sum of the four marker PAHs (benzo(a)pyrene, benzo(a)anthracene, benzo(b)fluoranthene, and chrysene) for smoked meats at the level of 5 µg/kg and 30 µg/kg, respectively.

The PAH content in smoked products is dependent on many factors, including the temperature and time of smoking, type of heat source, size and fragmentation of the product, type of casing, level of curing, additional smokehouse equipment, the passage of smoke through the chamber, type and dampness of the wood, cleaning of the smokehouse, and the amount of oxygen delivered to it [11,14]. The thermal treatment applied by smoking plants must ensure the safety of the smoked food and meet the requirements of the relevant legal acts regarding the maximum PAH levels in smoked meat products [10,13,15]. That is why PAH-content monitoring in traditionally smoked meat products is recommended, as the available scientific data indicates [6,11].

Considering the above, the work aimed to analyze the PAH contamination levels in Polish meat products smoked in traditional smokehouses with direct and indirect heat sources and in an industrial way as an element of PAH-content monitoring of market products and refer them to the applicable legal limits in this area. An important aspect of the research was to link several technological factors used in the production of the analyzed products with the levels of their contamination and, based on the statistical analysis of the data, to propose specific strategies for reducing PAH levels in smoked meat products for producers.

## 2. Materials and Methods

### 2.1. Research Material

This research material consisted of 12 smoked meats (W) and 38 smoked sausages (K), medium or coarsely minced (Table 1). The products were obtained directly from seven producers from 2022 to 2023. The plants were approved or registered at the Veterinary Inspection in Poland. The products analyzed were made from pork meat. The pigs were raised in Poland. Samples for testing were taken following the principles specified for the purposes of official control of the PAH levels in foodstuffs [9]. The bulk sample of each product weighing at least 1 kg consisted of primary samples, the number of which depended on the size of the production batch of a given smoked product. In the case of batches below 50 kg, 3 samples were taken. From batches weighing 50 to 500 kg, 5 samples were taken, while if the production volume was above 500 kg, 10 samples were taken. The mass of the primary sample was at least 100 g. Primary samples were taken from the part under and above the smoking stick, thus obtaining a representative bulk sample. The bulk samples were wrapped in aluminum foil and labeled with the product name, manufacturer’s name, batch number, and expiration date, and then transported to the laboratory under refrigerated conditions.

The criteria for selecting products for testing was the observed increase in sales of products from two technological groups: medium-ground sausages and smoked meats, the production volume of a given assortment in the plants, and the highest probability of PAHs in smoked products based on scientific literature and the results of internal and external studies conducted by the production plants. The plants whose products were sampled for testing agreed to provide the technological factors used in producing the analyzed samples of meats. The list of analyzed products and technological factors used in their production are presented in Table 1.

The tested cold cuts (sausages and smoked meats) were smoked in traditional smoking chambers (traditional smoking) or smoking and steaming chambers with a smoke generator (industrial smoking). The source of smoke in traditional smoking chambers was burnt pieces of wood from deciduous trees (alder, beech, oak, and/or wild cherry). The wood was barked or unbarked. Products suspended on smoking sticks were placed directly above the hearth (direct smoking) or at a distance from it (indirect smoking). For industrial smoking, smoking and steaming chambers with a smoke generator were used, in which the source of smoke was burnt beech and alder chips. Several products were smoked in smoking and steaming chambers with a smoke preparation applied to the products by the atomization method.

The products were smoked for 30 to 420 min at a temperature of 50 to 100 °C. Natural spices (e.g., salt, pepper, garlic, bay leaf, allspice, cloves, and rosemary) or ready-made mixtures of spices were used in the products. The sausages were produced in natural pork, mutton, and protein casings. Some of the analyzed products were dried before smoking. The frequency of cleaning the smoking chamber was also a technological factor. Some smokehouses had valves, fans, shafts, and chokes (Table 1).

### 2.2. Analytical Methods

#### 2.2.1. Chemicals and Materials

Acetonitrile (HPLC gradient grade), acetone, cyclohexane, and ethyl acetate (all of analytical purity > 99.9%) from Chemsolve were provided by Witko (Łódź, Poland) and from Chempur by Chemilab (Tarnobrzeg, Poland). Original Quechers Extract Pouches (sodium chloride, anhydrous magnesium sulfate) of Agilent Technologies were purchased from Altium (Warsaw, Poland). Deionized water was obtained from an ELGA PureLab Classic water purification system. Centrifuge tubes (50 mL) of BIOFIL were provided by Alchem Grupa Sp. z o.o. (Toruń, Poland).

For the analysis, acetone solutions of a mixture of native PAHs: benzo(a)anthracene, chrysene, benzo(b)fluoranthene, and benzo(a)pyrene (Dr. Ehrenstorfer GmbH, Augsburg, Germany) and deuterated PAHs: benzo(a)anthracene-D12, chrysene-D12, benzo(b)fluoranthene-D12, and benzo(a)pyrene-D12 (Dr. Ehrenstorfer GmbH, Augsburg, Germany) were used.

#### 2.2.2. Sampling for Analysis

The samples were taken for analysis following the sampling rules for the official control of the levels of PAHs in foodstuffs. They were collected from the lower part, where the PAH content is the highest, and from the part above the smoking stick, thus obtaining a representative aggregate sample. The bulk sample weight was approximately 1 kg. Collective samples were wrapped in aluminum foil and transported to the laboratory under refrigeration conditions [9].

The research was carried out for 4 years, taking samples from plants under the supervision of the Veterinary Inspection. All tests were performed in an accredited laboratory. The analyses were conducted using gas chromatography with mass spectrometry (GC-MS). The results are given with expanded uncertainty at the significance level of 95% and coverage factor k = 2.

#### 2.2.3. PAH Content Analysis

The PAH content analysis was based on the methodology of Niewiadowska et al. [16] with some modifications. The analysis consisted of determining the content of 4 marker PAHs: benzo(a)anthracene—B(a)A; chrysene—Chr; benzo(b)fluoranthene—B(b)F; and benzo(a)pyrene—B(a)P by gas chromatography with mass spectrometry detector (GC-MS), in the range of 0.9 µg/kg up to 100 µg/kg in traditionally smoked meat products, as well as those smoked in smoking and steaming chambers.

##### Determination of PAHs Using the GC-MS Method

An amount of 3.0 g of the previously finely ground sample was weighed into a 50 mL centrifuge tube. Then, 0.45 mL of the working solution of the internal standard, Mix PAH-D12 with a concentration of 0.1 µg/mL, was added. After 30 min, 12 mL of distilled water was added and mixed thoroughly in a shaker for 3 min. Then, 15 mL of acetonitrile was added to the tube and mixed. Subsequently, QuEChERS salts (6 g of magnesium sulfate and 1.5 g of sodium chloride) were added and mixed again for 3 min. The sample was centrifuged for 30 min in an Eppendorf Centrifuge 5804R (Hamburg, Germany) for phase separation at 3500 rpm at −9 °C. After centrifugation, 8 mL of the extract was taken and evaporated to dryness in a stream of nitrogen at 40 °C. Then, the residue was dissolved in 2 mL of cyclohexane.

The SPE columns were placed in the solid-phase extraction setup, and then each column was conditioned by washing it with 1 mL of cyclohexane. The extract dissolved in cyclohexane was applied to the column. The column was then washed with 1 mL of cyclohexane. The analyzed analyte was eluted thrice using 1 mL of ethyl acetate. The eluate was evaporated to dryness at 40 °C under a stream of nitrogen.

The residue was dissolved in 0.5 mL of acetone and transferred to a chromatography vial, and finally, PAH determination was conducted using the Agilent GC/MS 6890N/5975B gas chromatograph with a 7693 autosampler (Longwood, FL, USA). The operating conditions were as follows: Rxi-PAH 40 m × 0.18 mm × 0.07 μm capillary column with midpolarity proprietary phase, catalog no. 49316 (Restek, Benner Cir, Tuskegee, AL, USA); helium was used as the carrier gas at 1.0 mL/min; the injector temperature was set to 325 °C in the splitless injection mode with a 2 µL injection volume. The ion source and interface temperature were maintained at 300 and 320 °C, respectively. The GC oven temperature program was as follows: 80 °C for 0.5 min, ramping from 80 °C to 200 °C at 40 °C/min, ramping from 200 °C to 210 °C at 2.3 °C/min and holding at 210 °C for 4 min, ramping from 210 °C to 260 °C at 3 °C/min and holding 260 °C for 5 min, followed by an increase to 320 °C at 10 °C/min and holding 320 °C for 13 min. The quadrupole analyzer measured ion abundances in the *m/z* range of 50 to 450, with a detector voltage of 1.5 kV. Electron ionization (70 eV) was employed with a selected ion monitoring (SIM) mode.

The extracts were dosed into the chromatograph, starting with standard samples—calibration solutions from the concentration of PAH solution of 0.9 µg/kg to 100 µg/kg, then the reagent sample, test samples, and PAH standard solution, with a concentration of 2 µg/kg as a control sample.

##### Qualitative–Quantitative PAH Analysis

The calibration curve was prepared based on the analysis of standard solutions of PAHs in acetone, with the following concentrations: 0.9, 2, 5, 25, 50, and 100 µg/kg.

To prepare a control sample with the standard addition, 0.45 mL of the working solution of the internal standard at the concentration of 0.1 µg/mL Mix PAH-D12 and 0.06 mL of the solution of native PAH Mix at the concentration of 0.1 µg/mL were added to the sample solution based on the meat matrix without PAHs. Subsequently, the same procedure was followed for the proper samples.

PAH identification was conducted in the SIM mode, measuring each compound’s two most abundant ions from the molecular ion cluster and based on comparing the GC retention time with PAH standard solutions and the characteristic ions and their percentages in the spectrum monitored during the analysis. Ions analyzed for each PAH in the SIM mode were as follows: B(a)A—228, 226; Chr—228, 226; B(b)F—252, 250; B(a)P—252, 253. The relative intensity of the detected ions, expressed as a percentage of the intensity of the most intense ion, corresponds to the intensity of the standard solutions of the samples enriched with the analyte under testing, measured under the same conditions and within the tolerance limits specified in Table 2.

In PAH quantification, the concentration of the analyte in the test sample in µg/kg was calculated based on the comparison of the ratio of the peak area of the analyzed analyte—PAHs to the peak area of the PAH-D12 internal standard and by referring this value to the calibration curve. The final result of the PAH content determination was the arithmetic mean of three parallel repetitions for each of the samples not differing by more than 0.50 μg/kg for B(a)A; 0.45 μg/kg for Chr; 0.42 μg/kg for B(b)F; and 0.54 μg/kg for B(a)P. The results were given following the requirements of Commission Regulation (EU) 836/2011 [9], as X ± U, where X is the analytical result, and U is the expanded measurement uncertainty.

#### 2.2.4. Method Validation

The following validation parameters were tested: the method linearity range, the limit of detection (LOD), the limit of quantification (LOQ), recovery values, and the method precision.

The linearity range of the method was confirmed in the range from 0.9 µg/kg to 100 µg/kg. The LOD for the analyzed compounds was 0.3 µg/kg, while the LOQ was 0.9 µg/kg. In the recovery tests, smoked meat samples were fortified with four PAH concentrations (0.9, 2.0, 5.0, and 100 µg/kg). The recovery values for the analyzed compounds ranged from 65 to 109%. The recovery values obtained are within the recommended range of 50% to 120%, regarding the criteria required for the PAH determination methods by Commission Regulation (EU) No. 836/2011 [9]. The remaining validation parameters also meet the requirements of this regulation. The method’s precision, determined as the coefficient of variation (CV) under the repeatability conditions of the method for all four fortification concentrations analyzed, ranged from 2.0 to 14.8%, which is also within the required criterion of CV ≤ 20%. Therefore, satisfactory validation parameters following the legal requirements were obtained for the applied method of PAH content determination.

### 2.3. Statistical Analysis

The results were statistically analyzed using Microsoft Excel v 2013 and STATISTICA 13 PL. To determine the relationship between selected technological factors and the content of PAHs, principal component analysis (PCA) was carried out together with classification and cluster analyses.

## 3. Results and Discussion

### 3.1. PAH Content in Smoked Meats

In the traditionally smoked products analyzed in this study, the applicable PAH limits were not exceeded, regardless of the smoking method, product diameter, type of spices used, or smoking time. The content of benzo(a)pyrene in all ungrounded traditionally smoked products did not exceed the LOQ (<0.9 µg/kg) (Table 3). Only one ungrounded product (W8) from those analyzed in this study was smoked in a traditional smoking chamber with an indirect hearth using seasoned wood. The indirect smoking method is considered safer for the consumer compared to placing the smoked product directly above the hearth, as confirmed by Semanova et al. [3], Zachara and Juszczak [17], and Sampaio et al. [18].

Among the examined traditionally smoked meats, only in one sample (W7) was the sum of four marker PAHs above the limit of quantification, amounting to 2.5 ± 0.6 µg/kg. The product was smoked directly over the hearth. Natural spices were used in its production. The smoking time was the longest of all the directly smoked products analyzed and ranged from 90 to 120 min. The diameter of the product was one of the lowest in this group and amounted to 60–120 mm. The higher content of the sum of the four PAHs may result from the direct method of smoking, long smoking time, high smoking temperature (70–80 °C), the use of unseasoned and barked wood, as well as from less frequent cleaning of the smokehouse (twice a month) compared to other products smoked directly over the hearth (Table 3).

Four analyzed samples (W9–W12) were smoked industrially. The contents of B(a)P and the sum of the four PAHs in these samples were below the LOQ.

The analyzed smoked meat samples were produced in different conditions, both in terms of smoking time and temperature, smoking method (traditional direct or indirect method and method in chambers with a smoke generator—industrial), the wood used, type of smoke, additional equipment, as well as diameter, or the use of an additional process, e.g., drying or steaming. Despite the different technological factors, in all analyzed samples, the contents of B(a)P and the sum of the four PAHs followed legal regulations. In all analyzed samples, except for sample W7, the determined values were below the LOQ. It can, therefore, be concluded that the sample diameter (large) and lack of fragmentation are factors that have a significant impact on the generation of PAHs in smoked products.

Roseiro et al. [19], in their studies concerning contamination of Portuguese traditional dry-smoked meat products manufactured in micro industrial plants, stated that among all products processed under industrial conditions, the B(a)P limit was never exceeded, and about 81% of the samples presented a B(a)P content lower than 1.0 μg/kg. Similarly, Škaljac et al. [20] proved the safety of traditionally smoked meat products originating from north Serbia, including dry-cured meat products, bacon, and dry-fermented sausages, regarding the PAH limits established by Commission Regulation (EU) No. 915/2023 [10], since the B(a)P contents were below the LOD in all examined traditional meat products while the contents of the four marker PAHs were much lower than the set maximum value (0.057–2.22 µg/kg). The cited conclusions are consistent with those obtained in our study of smoked meat samples. In turn, Kafouris et al. [21] determined significantly higher concentrations of benzo(a)pyrene and the sum of the four PAHs than in our study, which was associated with a long smoking time, conducted several times a week for 3–4 months, which confirms the effect of smoking time on the PAH content in smoked products. Tsutsumi et al. [22] analyzed smoked meat products in Japan and determined the content of B(a)P at the level from 0.036 µg/kg to 0.58 µg/kg, and the sum of the four PAHs from 0.17 µg/kg to 5.2 µg/kg. Puljić et al. [23] stated that industrially produced Buđola samples (dry-cured pork neck originating from Herzegovina) are safer for consumption than the product smoked in traditional smokehouses in uncontrolled conditions. The B(a)P content levels were undetectable in the samples analyzed, and the sums of the four marker PAHs in all industrially smoked samples were below the quantification limit. However, in traditional Buđola samples, the mean four PAH levels were equal to 28.77 µg/kg in the surface layers and 21.14 µg/kg in the inner layers.

The content of PAHs in smoked meats has been the subject of many studies published in recent years. The authors focused, among others, on indicating the factors that most generated the formation of PAHs in the analyzed products.

According to Bogdanovic et al. [24], Sojinu et al. [25], Onopiuk et al. [26], Wang et al. [27], and Ciecierska et al. [28], PAH formation depends on many factors, including the method of smoking, the temperature, the distance of the smoked product from the hearth, the type of wood used for smoking, the duration and temperature of smoking, the fat content of the smoked product, the type of product, and the cleanliness of the smokehouse. Also, Ledesma et al. [1] and Racovita et al. [29] identified the above variables as factors needing control to minimize PAH concentrations in smoked products. In addition to the above parameters, Ledesma et al. [1], Sampaio et al. [18], Onopiuk et al. [26], and Iko Afe et al. [30] also indicated the construction of the smoking chamber and its equipment as factors affecting the PAH content in smoked products. According to Puljić et al. [31], the most important factors influencing the level of PAHs in smoked products are the type of smoking, the type of wood used, the smoking time, and the type of smoked food. In turn, Malaruta and Vagnaia [32] indicated the type of wood as the most important factor influencing the content of PAHs. Studies by Ledesman et al. [1] and Zelinkova and Wenzel [33] confirm that direct smoking and hot smoking result in a higher level of PAH contamination of meat products than the indirect method. The results obtained in the group of smoked meats below the LOQ make it impossible to conclude the relationship between the diameter of the product and the level of PAHs.

The determined contents of B(a)P and the sum of the four marker PAHs in smoked meats did not exceed the maximum levels allowed by the applicable legal regulations (2.0 µg/kg and 12 µg/kg, respectively) [10]. It can, therefore, be considered that these products are safe for the consumer’s health.

### 3.2. PAH Content in Smoked Sausages

Much more varied results were obtained when analyzing sausages. Out of 38 samples, the B(a)P contents were below the LOQ in nine products (K1, K2, K11, and K33–K38).

The content of B(a)P and the sum of the four marker PAHs for coarsely minced sausages traditionally smoked directly over the furnace did not exceed the applicable limits. Two samples, K1 and K2, were determined at a level below the LOQ. These products were smoked for 60 min. They were characterized by a larger diameter than the other sausages analyzed (80 and 50 mm, respectively). They were placed in artificial casings, which, as confirmed by research, show better barrier properties to smoke penetration and, as a result, limit the penetration of B(a)P into the product, contributing to a reduction in the content of this compound in smoked meat products [1,8,28]. Thus, the type of casing has a significant impact on PAH contamination in traditionally smoked meat products. In addition, removing the casing before product consumption is recommended to reduce exposure to PAHs [26,28].

For comparison, in samples K13–K16, which were also smoked for 60 min using the traditional direct method but characterized by a small diameter (28–32 mm), the content of B(a)P was determined at a level from 2.7 to 6 µg/kg, and the sum of the four PAHs at a level from 6.2 to 13.7 µg/kg. It is worth noting that among the tested coarsely ground sausages, both the level of B(a)P and the sum of the four marker PAHs was the highest in the samples of coarsely ground sausage (K3) steamed in a natural casing and smoked in a traditional smokehouse with an indirect heat source and amounted to 6.8 ± 1.8 μg/kg for B(a)P and 10.6 ± 2.5 μg/kg for the sum of the four PAHs, despite the largest product diameter of all coarsely minced sausages (120 mm) (Table 4).

The results indicate that the permissible limits of five traditionally smoked products belonging to the medium-minced sausage group are exceeded (K5, K12, K22, K29, and K30). In three of these samples (K10, K17, and K28), the level of B(a)P was equal to the maximum allowable limit (5 µg/kg) (Table 4). These sausages were smoked directly over the hearth at a temperature of 70–80 °C in dense smoke for 90–150 min. The relatively high level of B(a)P may be influenced by the direct method of smoking, high temperature, and long smoking time. The first and third of the analyzed products were smoked with oak or alder wood, and the second with oak wood. The sum of the four PAHs in these products did not exceed legal regulations’ permissible levels in force. Two samples (K5, K12) were characterized by a B(a)P content of 20.8 and 10.7 μg/kg, respectively, significantly exceeding the permitted standards. Both samples were smoked with wood, which was not always debarked and seasoned (Table 1). The information obtained from the establishment owner shows that the first of the analyzed sausages (K5) was dried in the smokehouse for 80 min and was the last product of the batch smoked in the smokehouse in hot smoke, which may have contributed to the high level of B(a)P in this sample. Despite the relatively short smoking time (90 min) compared to other medium-minced sausages, as well as the weekly cleaning of the smokehouse and additional equipment of the smokehouse with fans, dampers, gate valves, and chokes, the B(a)P level significantly exceeded the legal limit (20.8 ± 5.4 μg/kg). The smoking time of the second analyzed sausage (K12) smoked directly above the oven in hot smoke was long (120–150 min) compared to the other samples, which could have contributed to exceeding the B(a)P concentration, which was 10.7 ± 2.8 μg/kg. The content of PAHs could also be affected by washing the smokehouse twice a month, as well as the lack of additional smokehouse equipment and drying the meats before smoking in the smokehouse chamber for 30–60 min [1,18,24,26]. The exceeded B(a)P level in both samples could also be caused by the lack of additional post-smoking treatment (evaporation), which, according to the available literature, could reduce the contamination of the product with PAHs [1,34]. In both of the samples cited, the levels of the total four PAHs content were quite high and amounted to 31.5 ± 7.5 μg/kg and 37.7 ± 9 μg/kg, respectively. However, the marked levels aligned with the limit established by law for the products covered by the derogation (taking into account the measurement uncertainty) [15].

The highest level of B(a)P (20.6 ± 5.4 µg/kg) was determined in the K22 sausage, produced in a natural casing, smoked for 420 min with dense smoke at a temperature of 70–80 °C (Table 4). The reason for such a high content of PAHs, apart from the long smoking time, could also be the use of bark-free wood and the lack of additional treatment after the smoking process—steaming [1,24,26,31,34]. The remaining two assortments, K29 and K30, in which the permissible levels of PAHs were found to be exceeded, were smoked for 180–240 min. High levels of B(a)P, respectively, 10.1 ± 2.6 µg/kg and 12.1 ± 3.2 µg/kg, could have been caused by a rather high smoking temperature of 70–80 °C, rare cleaning of the smokehouse (once per month), the use of unbarked wood, and the drying of the product before smoking. The total level of the four PAHs determined in samples K22 and K30 (Table 4) was 35.5 ± 8.5 µg/kg and 32.2 ± 7.7 µg/kg, respectively, while in sample K29, it was 17.5 ± 4.2 µg/kg (Table 4). Exceeding the B(a)P content in the samples may also result from the spices and functional additives used in the production, which may be a source of PAHs [35,36,37,38]. The producers of the products analyzed in this study did not test the content of the PAHs in the spices added to their products. Therefore, it is impossible to refer to the obtained results and link the determined levels of PAHs with their content in spices. For the same reason, referring to the potential presence of PAHs in raw meat is impossible. According to Puljić et al. [31] and Mastanjević et al. [38,39,40], contamination of fresh meat of animals for slaughter may result from the contamination of soil, air, and water and their accumulation in plants eaten by animals as fodder, which allows them to be deposited in the muscles.

In none of the six industrially smoked medium-minced sausages (K33–K38) analyzed in this study, the PAH content exceeded the LOQ (<0.9 μg/kg). All products contained ready-made spice mixes and were smoked in less than an hour. Beech and alder chips were used to smoke all samples (Table 4).

The research by Fasano et al. [41] determined the content of benzo(a)pyrene from 3.1 to 98 µg/kg and the sum of the four PAHs from 38 to 1367 µg/kg in Spanish garlic sausage, which significantly exceeded the results obtained in this study. The products analyzed by the authors were smoked traditionally and were present in natural casings. In turn, da Silva et al. [42], in 55 sausages purchased in Brazil, determined the concentration of B(a)P and the sum of the four PAHs at levels ranging from below the LOQ to 2.44 µg/kg and from below the LOQ to 48.98 µg/kg, respectively. Only two sausages showed a B(a)P content above the permissible limit, while for six sausages, the four PAH concentrations above the legal limit of 12 µg/kg were noted.

According to Fasano et al. [41], approximately 90% of PAHs are deposited on the casings. However, Mastanjević et al. [39] showed that, apart from the type of casing, an important factor generating PAHs is the distance of smoked products from the hearth. Puljić et al. [31] showed higher levels of PAHs in the external parts of smoked meats, both traditionally and industrially. In turn, Ledesma et al. [1,34] found that additional treatment after smoking, such as rinsing the smoked product or immersing it in water, helps to remove soot and PAHs from the surface of the smoked food. The high content of PAHs may also result from the type of wood used, which was confirmed by Sampaio et al. [18], Mastanjević et al. [40], and Alsadat Mirbod et al. [43]. The last-mentioned researchers found the lowest content of the four PAHs in sausages smoked with poplar wood, ranging from 4.3 ± 0.4 to 8.1 ± 0.8 µg/kg [43]. Racovita et al. [29] showed a correlation between the higher content of lignin contained in wood and the higher amount of PAHs in the smoked product, and also almost five times higher content of PAHs in sausages smoked using plum wood compared to beech wood. The authors also confirmed the effect of smoking time on the content of PAHs in smoked products, indicating that extending the smoking time from 2 to 6 h resulted in a three-fold increase in the level of B(a)P and a two-fold increase in the sum of the four PAHs in traditionally smoked sausages. The relationship between longer smoking time and higher smoking temperature and higher PAH accumulation in smoked products is also indicated by Palade et al. [8], Kafouris et al. [21], Puljić [23], Hokkanen et al. [44], Fraquez et al. [45], and Lu et al. [4]. Palade et al. [8] showed a relationship between the higher water content and lower PAH concentration in the product. In turn, Škaljac et al. [20] determined the content of B(a)P and a total of the four PAHs in sausages below the LOQ, except for one sample (K5), in which the sum of the four PAHs was 1.8 µg/kg. The sausages analyzed by the authors differed in diameter (26–30 mm, 55 mm, 80–90 mm), casing (collagen or natural), and smoking time (from 3 to 7 days, for 4–12 h per day), and two samples were smoked with beech-cherry wood (the others only with beech wood). Despite the different technological factors, in all analyzed samples, the content of the determined compounds was below the LOQ, which confirms the complexity of the smoking process and the influence of many factors on PAH formation in this process. In turn, Onopiuk et al. [26] indicate the influence of the position of the smoked product in the smoking chamber as one of the important parameters of the smoking process. Products placed in the middle and high contain more PAHs in the outer layers than those placed at the lowest level in the smokehouse. In turn, products placed on the lowest level of the smokehouse are characterized by the highest content of PAHs inside the product.

In conclusion, based on the analyses and the results obtained, it was found that the PAH content in traditionally smoked products is much higher compared to industrial smoking, which was also shown by Puljić et al. [31] and Zelinkova and Wenzl [33]. Differences in the PAH contamination levels most likely result from greater control of the parameters of the smoking process in the industrial method. The application of good smoking practices, consisting of the use of barked and seasoned wood, additional smokehouse equipment, and proper hygiene of the smokehouse, enable the PAH levels in traditionally smoked products to follow the limits set out in the legal regulations. Only in one smoked meat subjected to traditional smoking (W7) did the total content of the four PAHs exceed the permissible limits (2.5 ± 0.6 µg/kg). The content of PAHs in products from the group of smoked meats and medium-minced industrially smoked sausages did not exceed the LOQ. On the other hand, the determined concentration of PAHs in medium-minced traditionally smoked sausages ranged from 1.3 ± 0.3 µg/kg in sample K26 to 20.8 ± 5.4 µg/kg in sample K5 for B(a)P and from 4.7 ± 1.1 µg/kg in the K32 sample to 37.7 ± 9 µg/kg in the K12 sample for the total four PAHs’ contamination.

The results obtained for traditionally smoked meat products confirm the values of the PAH monitoring program commissioned by the Chief Veterinary Officer in Poland and the report on the project implemented by the CDR (Center for Agricultural Advisory) in Brwinów, Radom Branch in 2014 [38,46]. About 5% of samples of traditionally smoked meat products exceeded the B(a)P limit, and the limit of the four PAHs was exceeded in about 8% of the tested products, mainly sausages.

The higher level of PAH content in traditionally smoked sausages compared to industrially smoked sausages, as well as the higher level of PAH in sausages compared to smoked meats (not crushed), was confirmed by Ciecierska and Obiedziński [47], Zachara et al. [11], and Bogdanović et al. [24], which resulted from a larger absorptive surface with a smaller diameter of the product. Rozentale et al. [46], Bogdanović et al. [24], and Onopiuk et al. [26] also found that the higher the surface area to weight ratio of smoked products, the higher the PAH content.

Analyses of the variables affecting the content of PAHs in smoked meat products indicate that the contamination of smoked meat products can be reduced by controlling the smoking process [34,37]. Important technological factors helping to reduce the level of PAHs include, among others, the use of the indirect method of smoking; reduction in smoke temperature; use of appropriate wood; an increase in the distance of the product from the heat source; use of an external smoke generator; use of fans and filters; installation of devices to collect fat and prevent it from dripping onto the fire; use, where possible, the appropriate casings; and also the selection of high-quality raw material, especially with a low-fat content [28,48,49,50]. According to Domingo et al. [2], meat and meat products are not the main sources of PAHs among food products. Exposure to PAHs from food depends on the dietary preferences, type and frequency of consumed products, and the size of meals.

Summarizing the obtained results of the B(a)P content and the sum of the four PAHs, it should be stated that all analyzed products met the legally specified limits among the smoked meats. The situation was different in the case of sausages. The analysis of the B(a)P levels showed an exceedance of the permissible limits of these compounds in 13 out of 38 analyzed samples. However, considering the measurement uncertainty, the number of “non-compliant” sausages is smaller. Namely, sausages with the symbols K3, K10, K14, K17, K19, K21, K23, and K28, after taking into account the measurement uncertainty, were characterized by the B(a)P content below 5 µg/kg, following the legal limit. This results from the provisions of the legal act, based on which research laboratories and supervisory bodies are guided by the principle of trust in the entrepreneur [10,51]. When providing analysis results, they take into account, where appropriate, the measurement uncertainty. Based on this legal provision, the content of B(a)P in the cited samples should be considered as compliant with the limits contained in legal regulations. Similarly, considering the act’s provisions, the total content of the four PAHs in sausages K5, K12, K22, and K30, after considering the measurement uncertainty, is consistent with the legally adopted limits for these compounds. Taking into account the limits specified in the legal regulation [10], together with the principle of trust in the entrepreneur, samples marked with codes K5, K12, K22, K29, and K30 did not meet the requirements for the permitted content of B(a)P in smoked meat products.

### 3.3. Principal Component Analysis (PCA)

**Figure 1:**
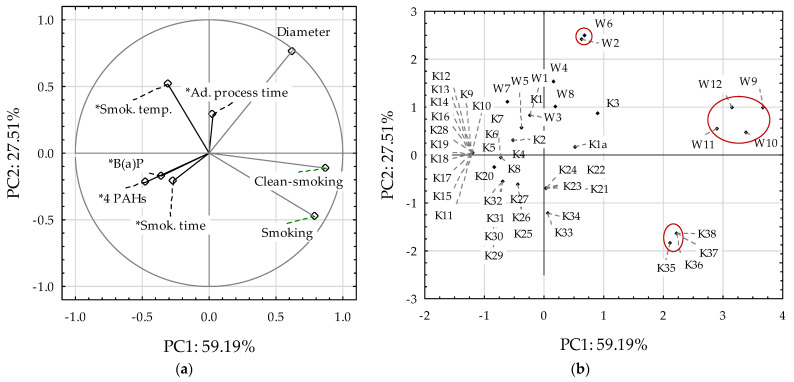

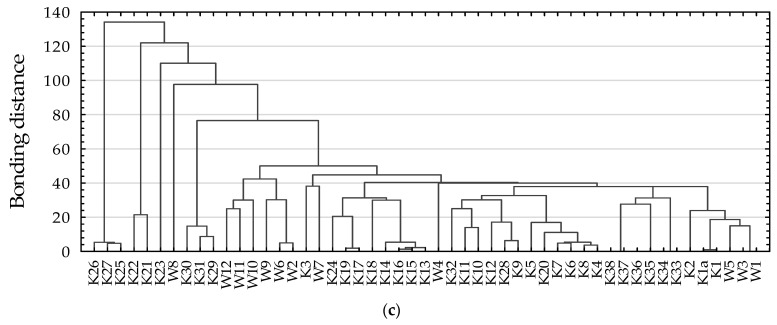
PCA and cluster analysis: (**a**) PCA loading plot of two principal components, (**b**) score plot presenting analyzed samples in terms of PC1 vs. PC2, and (**c**) cluster analysis. The red line on (**b**) concerns the separation of a group of data with a similar effect of factors involved in the smoking process.

Principal component analysis (PCA) and cluster analysis were applied to discuss the results (Figure 1). This allowed for a comprehensive determination of the relationship between the B(a)P content, the total content of the four marker PAHs, and the factors taken into account in the smoking process (Figure 1). The main components PC1 and PC2 explained 86.7% of the dependence of the PAH content in smoked products on technological factors. Due to the nonparametric nature of many technological factors for PCA and cluster analysis, six factors, including the smoking method (1—traditional, direct; 2—traditional, indirect; and 3—in the smoking and scalding chamber), smoking time (minutes), smoking temperature, product diameter, and additional process time (minutes) were used.

The smoking method, cleaning the smoking chamber (cleaning—smoking), and the sample diameter had the highest impact on the formation of the main components (Figure 1a). The greatest influence on the formation of the PC1 component was exerted by the smoking method, cleaning of the smoking chamber (cleaning—smoking), and the sample diameter, while on the PC2 component, it was the smoking temperature (Figure 1a). No high correlations were found between the indicators studied. Among the highest values, there was a negative correlation between the smoking method and smoking temperature (r = −0.640) and a positive correlation between the smoking method and the cleaning of the smoking chamber (r = 0.564). This is an important relationship. However, based on the previously discussed data on many other indicators not included in the PCA analysis, it is difficult to indicate a specific smoking method here. The smoking of the scalding chamber method did not generate PAH components; their content did not exceed 0.6 µg/kg. Occasionally, more significant amounts of these compounds, reaching 20.8 µg/kg B(a)P and 37.7 µg/kg for the four PAHs, appeared using traditional methods, more often with the direct method. Therefore, considering many other technological indicators, it can be assumed that both the conventional method, although indirect, and the currently used in the scalding chambers have their justification for use. It should be emphasized that there is a reasonably strong correlation (r = 0.509) between smoking time and distance of product (r = 0.474) with the content of B(a)P and the four PAHs compounds, the content of which increased with the extension of the duration of this treatment and the diameter of the products. It should be noted that the smoking temperature in the range of 65–85 °C was not as important as the smoking time in the 30–360 min range. However, additional smoking time had no significant effect on the PAH content. Therefore, it is suggested that higher temperatures and shorter times be used, as well as larger pieces for smoking.

The smoked meat W1–W12 samples were located in the positive part of PC2, creating two distinctive groups of samples with similar features. Smoked products W2 and W6, as well as W9–W12 were located next to each other, which confirms that these are products with similar values to the analyzed indicators.

Smoked meats W2 and W6 were produced using the traditional direct method under the same time and temperature conditions of the process. Both products were dried before smoking. In turn, smoked meats W9–W12 were obtained in a smoking-steaming chamber (referred to as industrial smoking). A spice mixture was added to the samples. The products were smoked at the same temperature. Sample W9 was smoked twice as long as the other mentioned smoked meats.

In the case of K1–K38 sausages, the samples were located mainly in the negative part of PC1, and among them, the K35–38 samples were in a separate, distinctive group. These sausages were smoked industrially, with the addition of a mixture of spices, at the same time and under different temperature conditions, except for sample K35, which was smoked at a higher temperature. The analyzed sausages were dried before smoking and had a similar diameter. Only sausage K35 was characterized by a significantly smaller diameter than the other samples in this group.

The first group consisted of sausage samples with a diameter of 30 mm, smoked traditionally indirectly for 360 min at 55 C (K25–K27), and containing 1.3–4.2 mg/kg B(a)P and 7.2–16.8 mg/kg of the four PAHs. These samples differed the most from samples W1, W3, W5, K1, and K2, with a diameter of 50–80 mm, smoked traditionally for 60–75 min at 75–85 C, and containing 0.6 mg/kg B(a)P and the four PAHs. The smaller diameter of the samples and longer time but lower temperature of sausage smoking using the traditional indirect method resulted in an increased content of PAH compounds, unlike the products smoked directly in a much shorter time but at a higher temperature.

## 4. Conclusions

The analysis showed significantly lower PAH contents in smoked meats than in sausages. In the group of smoked meats, except for one product, in all analyzed samples, the content of B(a)P and the sum of the four PAHs was below the limit of quantification while meeting the limits specified by law. In the group of medium-ground sausages, the analysis showed significantly higher levels of PAH contamination, which confirms the influence of the degree of grinding on the content of analyzed compounds. A higher fat content could also be the reason for higher contamination levels. The maximum benzo(a)pyrene content in some medium-ground sausages traditionally smoked using direct and indirect heat sources exceeded the maximum limit permitted by applicable law, confirming the smoking method’s influence on PAH contamination. The results also indicate that the natural casing is not a barrier against PAHs during traditional smoking, and a higher degree of fragmentation, together with a small cross-section, increases the PAH content in this technological group. Exceeded permissible limits of PAHs were recorded for products with natural spices and products with ready-made spice mixtures. Concentrations of benzo(a)pyrene exceeding the allowable standards were found in the case of sausages smoked for more than 60 min. However, the cluster analysis indicated the smoking time as a factor with a medium effect on the content of PAHs. It can, therefore, be concluded that the generation of PAHs in the product is a complex process and depends on many factors. Nevertheless, the research indicated time as one of the main determinants influencing PAH formation in the smoking process. An ambiguous effect of the wood used was shown, as was the frequency of cleaning the smokehouse on the content of PAHs in the analyzed products.

In some of the analyzed samples, the determined B(a)P level and the sum of the four marker PAHs exceeded the permissible limits set in Commission Regulation (EU) No. 915/2023. However, in accordance with the principle of trust in the manufacturer, in 12 out of 17 samples, after subtracting the measurement uncertainty, the level of the analyzed compounds followed the legal limits. As part of PAH levels monitoring in smoked meats and reducing exposure to these compounds via diet, production plants can be recommended to introduce changes within the framework of good manufacturing practices that reduce the PAH content in final products. The following recommendations serve as PAH reduction strategies and include, among others: changing, if possible, a direct firebox to an indirect one; shortening the smoking time or lowering the smoking temperature; reducing the amount or eliminating functional additives; replacing natural casings with artificial casings, including recommendations on the label to consume the product without a casing; drying the surface of products before the smoking process; using seasoned wood without bark for smoking, such as hornbeam, beech, ash, maple, elm, oak, acacia, alder, or fruit trees; loosely arranging pieces of wood in the firebox; using additional smokehouse equipment, such as fans and valves to regulate the air supply to the smokehouse as well as smoke extraction, and dampers and sheet metal plates to prevent fat from dripping onto the firebox, thanks to which it is possible to regulate the amount and density of smoke and maintain the correct temperature in the smoking chamber, and thus reduce the amount of PAHs; steaming and/or rinsing the smoked product with water; increasing the frequency of cleaning/washing the chamber of smokehouse; and installing a valve that separates the smoked product directly above the fireplace from the combustion zone.

Monitoring the levels of PAHs and their derivatives in food is becoming increasingly important, resulting from the wide presence of PAHs in the human environment and food and the negative still-studied effects of PAH exposure. Determination of the PAH content in food and the risk assessment resulting from the toxic effects of these compounds were, are, and should continue to be the subject of research to present the current situation in this area and protect people from exposure to PAHs. Undoubtedly, smoked meat products are a common dietary choice, especially in Poland, and can be a significant source of PAHs, making further research on PAH contamination levels and effective reduction methods imperative. Such studies are crucial for advancing food safety standards and ensuring consumer trust and satisfaction.

## Figures and Tables

**Table 1:** List of analyzed products and technological factors.

Sample Codes	Grinding	Type of Smoking	Type of Spices	Type of Wood	Wood Seasoning	Wood Debarking	Frequency of Cleaning the Smoking Chamber [per Month]	Type of Casings	Product Diameter [mm]	Smoking Time [min]	Smoke Temperature [°C]	Application of an Additional Process	Additional Smokehouse Equipment
W1	S	1	N	B	Y/N	Y/N	4	lack	80	60–90	70–100/dense smoke	Drying (W) 60–90 min, steaming	Valves, fans, shafts, chokes
W2	S	1	N	B	Y/N	Y/N	4	lack	150–200	60–90	70–100/dense smoke	Drying (W) 60–90 min, steaming	Valves, fans, shafts, chokes
W3	S	1	N	B	Y/N	Y/N	4	lack	80	60–90	70–100/dense smoke	Drying (W) 60–90 min, steaming	Valves, fans, shafts, chokes
W4	S	1	N	B	Y/N	Y/N	4	lack	120	60–90	70–100/dense smoke	Drying (W) 60–90 min, steaming	Valves, fans, shafts, chokes
W5	S	1	N	B	Y/N	Y/N	4	lack	50−80	60–90	70–100/dense smoke	Drying (W) 60–90 min, steaming	Valves, fans, shafts, chokes
W6	S	1	N	B	Y/N	Y/N	4	lack	150–200	60–90	70–100/dense smoke	Drying (W) 60–90 min, steaming	Valves, fans, shafts, chokes
W7	S	1	N	O/A	Y/N	Y/N	2	lack	60–120	90–120	70–80/dense smoke	Drying (W) 90–120 min, steaming	Lack
W8	S	2	M	O/A	Y	Y/N	1	lack	100–140	180–240	50–60/rare smoke	Drying (W) 90 min, steaming	Chokes
W9	S	3	M	B-A	ND	ND	Each time after production	lack	180	60	60–70/dense smoke	Drying (W) 90 min, steaming	Lack
W10	S	3	M	B-A	ND	ND	Each time after production	lack	150	30	60–70/dense smoke	Drying (W) 90 min, steaming	Lack
W11	S	3	M	B-A	ND	ND	8	lack	150	30	60–70/dense smoke	Drying (W) 120 min, steaming	Lack
W12	S	3	M	B-A	ND	ND	8	lack	150–200	30	60–70/dense smoke	Drying (W) 120 min, steaming	Lack
K1	C	1	N	B	Y/N	Y/N	4	O (protein casing)	80	60	70–80/dense smoke	Drying (W) 80 min, steaming	Valves, fans, shafts, chokes
K2	C	1	N	B	Y/N	Y/N	4	O (protein casing)	50	60	70–80/dense smoke	Drying (W) 80 min, steaming	Valves, fans, shafts, chokes
K3	C	2	N	A	Y	Y/N	4	N	120	120	70–80/dense smoke	Drying (H) 120 min, steaming	Chokes
K4	M	1	N	B	Y/N	Y/N	4	N	28–32	90	70–80/dense smoke	Drying (W) 80 min	Valves, fans, shafts, chokes
K5	M	1	N	B	Y/N	Y/N	4	N	28–32	90	70–80/dense smoke	Drying (W) 80 min	Valves, fans, shafts, chokes
K6	M	1	N	B	Y/N	Y/N	4	N	28–32	90	70–80/dense smoke	Drying (W) 80 min	Valves, fans, shafts, chokes
K7	M	1	N	B	Y/N	Y/N	4	N	28–32	90	70–80/dense smoke	Drying (W) 80 min	Valves, fans, shafts, chokes
K8	M	1	N	B	Y/N	Y/N	4	N	28–32	90	70–80/dense smoke	Drying (W) 80 min	Valves, fans, shafts, chokes
K9	M	1	N	O/A	Y/N	Y/N	2	N	28–32	120–150	70–80/dense smoke	Drying (W) 30–60 min	Lack
K10	M	1	N	O/A	Y/N	Y/N	2	N	28–32	90–120	70–80/dense smoke	Drying (W) 30–60 min	Lack
K11	M	1	N	O/A	Y/N	Y/N	2	N	28–32	90–120	70–80/dense smoke	Drying (W) 30–60 min	Lack
K12	M	1	N	O/A	Y/N	Y/N	2	N	28–32	120–150	70–80/dense smoke	Drying (W) 30–60 min	Lack
K13	M	1	N	O	Y	Y/N	2	N	28–32	60	70–80/dense smoke	Drying (W) 120 min, steaming	Shafts
K14	M	1	N	O	Y	Y/N	2	N	28–32	60	70–80/dense smoke	Drying (W) 120 min	Shafts
K15	M	1	N	O	Y	Y/N	2	N	28–32	60	70–80/dense smoke	Drying (W) 120 min	Shafts
K16	M	1	M	O	Y	Y/N	2	N	28–32	60	70–80/dense smoke	Drying (W) 120 min	Shafts
K17	M	1	N	O	Y	Y/N	2	N	28–32	120	70–80/dense smoke	Drying (W) 120 min	Shafts
K18	M	1	N	O	Y	Y/N	2	N	28–32	90	70–80/dense smoke	Drying (W) 120 min	Shafts
K19	M	1	N	O	Y	Y/N	2	N	28–32	120	70–80/dense smoke	Drying (W) 120 min	Shafts
K20	M	1	N	B	Y/N	Y/N	4	N (sheep casing)	18	90	70–80/dense smoke	Drying (W) 80 min	Valves, fans, shafts, chokes
K21	M	2	N	A	Y	Y/N	4	N	28–32	420	70–80/dense smoke	Drying (H) 120 min	Shafts
K22	M	2	N	A	Y	Y/N	4	N	28–32	420	70–80/dense smoke	Drying (H) 120 min	Shafts
K23	M	2	N	A	Y	Y/N	4	N	28–32	300	50–60/dense smoke	Drying (H) 120 min	Shafts
K24	M	2	N	A	Y	Y/N	4	N	28–32	120	50–60/dense smoke	Drying (H) 120 min, steaming	Shafts
K25	M	2	N	O-W	Y	Y/N	2	N	28–32	360	50–60/rare smoke	Lack	Shafts
K26	M	2	N	O-W	Y	Y/N	2	N	28–32	360	50–60/rare smoke	Lack	Shafts
K27	M	2	N	O-W	Y	Y/N	2	N	28–32	360	50–60/rare smoke	Lack	Shafts
K28	M	1	N	O/A	Y/N	Y/N	2	N	28–32	120–150	70–80/dense smoke	Drying (W) 30–60 min	Lack
K29	M	2	M	O/A	Y	Y/N	1	N	28–32	180–240	70–80/dense smoke	Drying (W) 60 min	Chokes
K30	M	2	M	O/A	Y	Y/N	1	N	28–32	180–240	70–80/dense smoke	Drying (W) 60 min	Chokes
K31	M	2	M	O/A	Y	Y/N	1	N	28–32	180–240	70–80/dense smoke	Drying (W) 60 min	Chokes
K32	M	2	M	O/A	Y	Y/N	1	N	28–32	90–120	50–60/rare smoke	Drying (W) 60 min, steaming	Chokes
K33	M	3	M	B-A	ND	ND	1	N	28–32	30	60–70/dense smoke	Drying (W) 60–90 min, steaming	Lack
K34	M	3	M	B-A	ND	ND	1	N	28–32	30	60–70/dense smoke	Drying (W) 60–90 min, steaming	Lack
K35	M	3	M	B-A	ND	ND	Each time after production	N (sheep casing)	18	55	60–70/dense smoke	drying (W) 45 min, steaming	Lack
K36	M	3	M	B-A	ND	ND	Each time after production	N	28–32	30	60–70/dense smoke	Drying (W) 45 min, steaming,	Lack
K37	M	3	M	B-A	ND	ND	Each time after production	N	28–32	30	60–70/dense smoke	Drying (W) 45 min, steaming	Lack
K38	M	3	M	B-A	ND	ND	Each time after production	N	28–32	30	60–70/dense smoke	Drying (W) 45 min, steaming	Lack

Grinding: S—not ground; M—medium-ground; C—coarsely ground. Smoking: 1—traditional, direct; 2—traditional, indirect; 3—in the smoking and scalding chamber. Type of spices: N—natural; M—mixture of spices. Type of wood: B—beech; O—oak; A—alder; W—wild cherry. Wood seasoning: Y—yes; N—no. Wood debarking: Y—yes; N—no. Type of casings: N—natural; O—another. An additional process: steaming and drying—in the production hall (H) and in the smoking chamber (W).

**Table 2:** Maximum allowable tolerance limits for relative ion intensities when using mass spectrometry technique.

Relative Intensity (% of Base Peak)	EI-GC-MS (Relative)
>50%	±10%
>20% do 50%	±15%
>10% do 20%	±20%
≤10%	±50%

**Table 3:** The content of benzo(a)pyrene and the sum of 4 marker PAHs in the analyzed smoked meats.

Sample Codes	Benzo(a)pyrene Content (µg/kg)	Measurement Uncertainty	Total Content of 4 PAHs (µg/kg)	Measurement Uncertainty
W1	<0.9	lack	<0.9	lack
W2	<0.9	lac	<0.9	lack
W3	<0.9	lack	<0.9	lack
W4	<0.9	lack	<0.9	lack
W5	<0.9	lack	<0.9	lack
W6	<0.9	lack	<0.9	lack
W7	<0.9	lack	2.5	0.6
W8	<0.9	lack	<0.9	lack
W9	<0.9	lack	<0.9	lack
W10	<0.9	lack	<0.9	lack
W11	<0.9	lack	<0.9	lack
W12	<0.9	lack	<0.9	lack

Code: W1–W12, smoked meats from different producers; <0.9—<LOQ.

**Table 4:** The content of benzo(a)pyrene and the sum of 4 marker PAHs in the analyzed smoked sausages.

Sample Codes	Benzo(a)pyrene Content (µg/kg)	Measurement Uncertainty	Total Content of 4 PAHs (µg/kg)	Measurement Uncertainty
K1	<0.9	lack	<0.9	lack
K2	<0.9	lack	<0.9	lack
K3	6.8	1.8	10.6	2.5
K4	2.4	0.6	10.4	2.5
K5	20.8	5.4	31.5	7.5
K6	3.3	1.2	18.9	3.9
K7	6.2	1.6	22.8	5.4
K8	4.3	1.1	13.6	3.2
K9	4.4	1.2	15.1	3.6
K10	6.8	1.8	17.9	4.3
K11	<0.9	lack	5.2	1.2
K12	10.7	2.8	37.7	9.0
K13	2.7	0.7	6.2	1.5
K14	6.0	1.6	13.7	3.3
K15	4.5	1.2	8.5	2.1
K16	4.8	1.2	7.2	1.7
K17	6.8	1.8	13.6	3.2
K18	3.2	0.8	5.3	1.3
K19	5.7	1.5	15.2	3.6
K20	2.7	0.7	12.4	3.0
K21	5.3	1.4	20.4	4.9
K22	20.6	5.4	35.5	8.5
K23	5.3	1.4	11.4	2.7
K24	3.5	0.9	11.5	2.7
K25	3.5	0.9	12.1	2.9
K26	1.3	0.3	7.2	1.7
K27	4.2	1.1	16.8	4.0
K28	6.7	1.7	21.0	5.0
K29	10.1	2.6	17.5	4.2
K30	12.1	3.2	32.2	7.7
K31	3.2	0.8	12.1	2.9
K32	1.6	0.4	4.7	1.1
K33	<0.9	lack	<0.9	lack
K34	<0.9	lack	<0.9	lack
K35	<0.9	lack	<0.9	lack
K36	<0.9	lack	<0.9	lack
K37	<0.9	lack	<0.9	lack
K38	<0.9	lack	<0.9	lack

Code: K1–K38, smoked sausages from different producers.

## Data Availability

The data presented in this study are available upon request from the corresponding authors.
